# Fungal Viruses Unveiled: A Comprehensive Review of Mycoviruses

**DOI:** 10.3390/v15051202

**Published:** 2023-05-19

**Authors:** Bianca Hough, Emma Steenkamp, Brenda Wingfield, David Read

**Affiliations:** Forestry & Agricultural Biotechnology Institute (FABI), Department of Biochemistry, Genetics & Microbiology, University of Pretoria, Pretoria 0002, South Africa; bianca.hough@up.ac.za (B.H.); emma.steenkamp@up.ac.za (E.S.); david.read@up.co.za (D.R.)

**Keywords:** mycoviruses, hypovirulence, hypervirulence, biocontrol, diversity, taxonomy, transmission

## Abstract

Mycoviruses (viruses of fungi) are ubiquitous throughout the fungal kingdom and are currently classified into 23 viral families and the genus *botybirnavirus* by the International Committee on the Taxonomy of Viruses (ICTV). The primary focus of mycoviral research has been on mycoviruses that infect plant pathogenic fungi, due to the ability of some to reduce the virulence of their host and thus act as potential biocontrol against these fungi. However, mycoviruses lack extracellular transmission mechanisms and rely on intercellular transmission through the hyphal anastomosis, which impedes successful transmission between different fungal strains. This review provides a comprehensive overview of mycoviruses, including their origins, host range, taxonomic classification into families, effects on their fungal counterparts, and the techniques employed in their discovery. The application of mycoviruses as biocontrol agents of plant pathogenic fungi is also discussed.

## 1. Introduction

Mycoviruses (viruses of fungi) are ubiquitous throughout the fungal kingdom [1]. They are known to associate with most of the major fungal taxonomic groups, including Ascomycota, Basidiomycota, Chytridiomycota, Zygomycota, and Neocallimastigomycota [2,3]. The International Committee on the Taxonomy of Viruses (ICTV) currently classifies mycoviruses as 23 families and 1 unclassified genus, and this is based on the type of genome [4,5,6,7,8]. The majority of mycoviruses have double-stranded RNA (dsRNA) or positive sense single-stranded RNA (+ssRNA) genomes [5]. Mycoviruses with dsRNA genomes are classified into the viral families *Chrysoviridae*, *Amalgaviridae Megabirnaviridae*, *Quadriviridae*, *Partitiviridae*, *Polymycoviridae*, *Reoviridae*, *Totiviridae*, and the genus *Botybirnavirus* (unclassified) [4,6]. Those with +ssRNA genomes are grouped into the families *Endornaviridae*, *Alphaflexiviridae*, *Barnaviridae*, *Deltaflexiviridae*, *Gammaflexiviridae*, *Hypoviridae*, *Narnaviridae*, *Mitoviridae*, *Hadakaviridae*, *Yadokariviridae*, and the reverse transcribing (RT) families *Metaviridae* and *Pseudoviridae* [4,6,7,8]. Mycoviruses with negative-sense single-stranded RNA (−ssRNA) genomes have also been discovered [9,10,11], and belong to the family *Mymonaviridae* [4,6]. Recently a number of ssDNA mycoviruses have also been found; however, only two belong to a recognized mycoviral family, namely Sclerotinia sclerotiorum hypovirulence-associated DNA virus 1 and Fusarium graminearum gemytripvirus 1 [12]. Both belong to the family *Genomoviridae* [13,14].

Mycoviruses that elicit hypovirulence have been identified in both human and plant pathogenic fungi. These viruses frequently cause several adverse effects in their fungal hosts, such as decreased virulence, irregular growth, abnormal pigmentation, and defects in sexual development [5,15,16]. The recent discovery of hypovirulence inducing mycoviruses in human pathogenic fungi presents an opportunity for the development of therapeutic interventions against fungal infections in humans. The majority of mycoviral research, however, has been concerned with hypovirulence-inducing mycoviruses of plant pathogenic fungi [12,17,18]. These mycoviruses have the potential to be used as biocontrol agents against their fungal hosts, thereby reducing the losses in agriculture and forestry due to fungal infections [19,20]. Despite their potential, the use of mycoviruses as biocontrol agents has a number of challenges. Mycoviruses lack the extracellular transmission mechanisms of plant and animal viruses [3]. Instead, viral transmission occurs intercellularly, through hyphal anastomosis (fusion of fungal hyphae), cell division, and sporulation [3]. Hyphal anastomosis impedes the successful transmission of mycoviruses between different fungal strains since they need to be vegetatively compatible with hyphal fusion [21]. Nevertheless, research is ongoing to find solutions to this problem [22,23], and mycovirus-based biocontrol has already been used successfully [19].

Advances in high throughput sequencing have led to a surge in mycovirus discoveries and new insights into their origins, diversity, and impact on fungal hosts. We review the latest developments in the field of mycovirology and evaluate the potential of mycoviruses as biocontrol agents of plant pathogenic fungi. We also summarize some of the methods which have been employed to help aid the discovery process.

## 2. The History and Origins of Mycoviruses

In 1948, a disease-causing dieback of the commercially produced mushroom, *Agaricus bisporus*, was reported and named the ‘La France’ disease [24]. It was not until nearly a decade later that mycoviruses were identified as the causal agents of this economically important disease [25]. This quickly led to the development of a new field of study: mycovirology. Not long after, mycoviruses were also identified in the ascomycete *Penicillium stoloniferum*, and were determined to cause interferon stimulation in mammals [26,27]. It was not until the 1970s, however, that a significant breakthrough was made in the field of mycovirology. A mycovirus of the chestnut blight pathogen, *Cryphonectria parasitica*, was observed to reduce the virulence of its host and had potential as a biocontrol agent against this plant pathogenic fungus [28]. This led to increased interest in the discovery of mycoviruses in plant pathogenic fungi, as they could serve as prospective biocontrol agents of such fungi. Another development during this period was the discovery of mycoviruses which induced the ‘killer yeast’ phenotype in *Saccharomyces cerevisiae*, a fungus that is routinely employed in winemaking, brewing, and baking [29]. These mycoviruses confer a competitive advantage to their fungal hosts by producing extracellular toxins which eliminate competing strains [30]. While these toxins were initially associated with fermentation failure, their potential use as a means of eliminating undesirable strains was soon realized [30].

Recent advances in high throughput technologies, specifically RNA sequencing, have facilitated the rapid discovery of mycoviruses. This has not only increased our understanding of mycoviral diversity within the fungal kingdom but has shed some light on the evolution and origins of these viruses. Two theories have been proposed. The ‘ancient coevolution theory’ posits that the relationship between mycoviruses and fungi is ancient and mirrors long-term co-evolution [3,5]. This is supported by the fact that mycoviruses lack an extracellular infection route, which complicates their transmission to other fungal species, and thus limits individual viruses to a single host [31]. Research by Neupane, et al. [32] also shows support for this theory, since the phylogenetic analysis of the RNA-dependent RNA polymerase (RdRp) sequences of mitoviruses from non-pathogenic arbuscular mycorrhizal fungi has revealed that they are highly conserved, and do not cluster with mitoviruses from other plant pathogenic fungi. The ‘plant virus theory’ on the other hand, suggests that mycoviruses originate from plant viruses, where they moved from the plant host to a fungus [3]. In support of this theory, studies have shown that mycoviruses often cluster with plant viruses on phylogenetic trees [33,34]. For example, Cryphonectria hypovirus 1–4 (CHV1-4), Fusarium graminearum virus 1 (FgV1), and Botrytis virus X are all related to plant potyviruses or potex-like viruses [5,35]. In addition, most mycoviruses lack a movement protein, and in some cases even a coat protein, which indicates that these viruses may have shed their non-essential genes to better adapt to their fungal hosts [31]. Natural cross-kingdom transfer of certain viruses between plants and fungi has also been demonstrated. Andika, et al. [36] provided evidence of this phenomenon by demonstrating the transmission of cucumber mosaic virus (CMV) from potato plants to *Rhizoctonia solani* through natural means. The transfer of plant viruses to fungi has also been shown by Cao et al. [37], where 11 different plant viruses were shown to transiently infect several plant pathogenic fungal genera, including *Alternaria*, *Lecanicillium* and *Sarocladium*. Cross-kingdom transmission has been proposed as a significant driver in the evolution of mycoviruses, given that the majority of mycovirus lineages have an ancestral link to plant viruses [38]. The opposite of this theory may also be true, where plant viruses may have originated from mycoviruses which moved from a fungus to a plant. For example, a recent study has demonstrated that mitoviral sequences can cross-transfer between *Botrytis cinerea* and cucumber plants [39]. Similarly, CMV has also been shown to move from *R. solani* to the plant host [36]. An alternative pathway for mycovirus transfer between fungi and plants, through the intermediary of a plant virus, has also been suggested. Bian, et al. [40] showed that CHV-1 was able to infect *Nicotiana tabacum* when co-inoculated with tobacco mosaic virus (TMV), a plant virus. Moreover, the findings suggest that CHV-1 may facilitate the accumulation of TMV in *Fusarium graminearum*, indicating a potential interaction between these viruses in a plant-fungal system. Due to a dearth of solid evidence and data, however, the exact origins of mycoviruses remains uncertain.

## 3. Diversity and Taxonomy

### 3.1. Fungal Host Range

Mycoviruses have been found across most of the major fungal taxonomic groups [1,5].

The majority of these mycoviruses associate with hosts from Ascomycota and Basidiomycota (collectively called Dikarya), as depicted in Table 1 and Table 2 [2]. However, this is most likely due to disproportionate sampling across the fungal kingdom. Recent studies have thus focused on the discovery of mycoviruses within the early diverging lineages of fungi and has led to the discovery of mycoviruses in Chytridiomycota, Zygomycota, Blastocladiomycota, and Neocallimastigomycota (Table 3) [2,41]. The ecological impacts of these viruses on their fungal counterparts, however, remains unknown and requires further investigation.

### 3.2. Mycoviral Taxa

**Figure 1:**
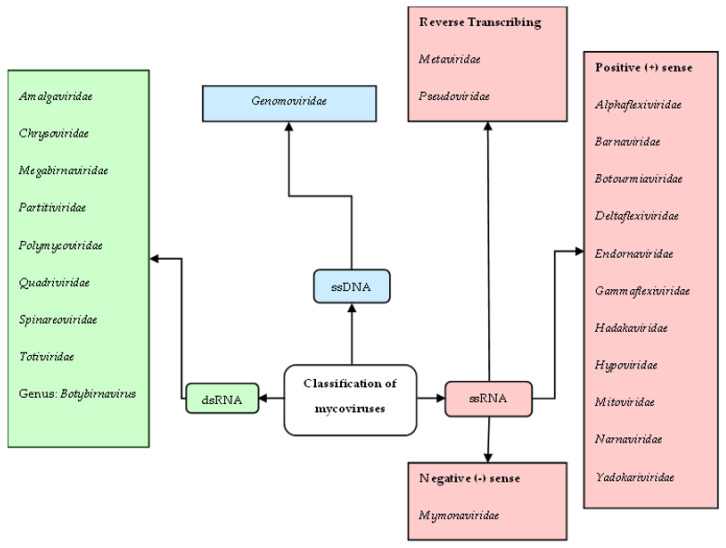
Mycoviral taxa currently recognized by the ICTV. Compiled using information from the official ICTV website (https://talk.ictvonline.org/, (accessed on 27 March 2023)).

Mycoviruses are currently classified into 23 families and one unclassified genus (Figure 1) by the ICTV according to their genome type and organization; https://talk.ictvonline.org/ (accessed on 27 March 2023). The majority of mycoviruses studied to date have dsRNA or ssRNA genomes, however, a few mycoviruses with ssDNA genomes have also been found [12,52,53,175,328].

#### 3.2.1. Double-Stranded RNA (dsRNA) Mycoviruses

The genome characteristics of mycoviruses, including size and segmentation, exhibit variation across families and genera. Specifically, the genomes of dsRNA mycoviruses can range from non-segmented, as observed in *Amalgaviridae* and *Totiviridae*, to bisegmented in *Megabirnaviridae*, *Partitiviridae*, and *Botybirnavirus*, quadripartite in *Quadriviridae*, or multisegmented in *Chrysoviridae*, *Polymycoviridae*, and *Spinareoviridae* [5,337,338,339,340,341,342,343]. Furthermore, genome size among dsRNA mycoviruses is highly diverse, with some families containing mycoviruses with genomes as small as 3.0 kb and others having members with genomes as large as 29 kb [338,339]. Detailed information on genome length and segmentation of dsRNA mycoviruses is provided in Table 4.

In addition to differences in genome size and segmentation, the genome organization of mycoviruses also varies among families and genera. Although the presence of an RNA-dependent RNA polymerase (RdRp) domain is ubiquitous among all mycoviruses, its location within the genome may differ [2]. For instance, the RdRp domain in multisegmented viruses of the *Chrysoviridae* family is encoded on the first genome segment, while in non-segmented viruses of the *Amalgaviridae* family it is located on the 3′ proximal open reading frame (ORF) [337,342]. With the exception of members of the *Botybirnavirus* genus, all dsRNA mycoviruses encode a capsid protein (CP) [5,337,338,339,340,341,342]. Additionally, some mycoviruses contain domains encoding proteins with unknown functions, as well as specific domains specific to certain families, such as the phytoreo S7 domain in the *Chrysoviridae*, a methyltransferase (Mtf) and proline-alanine-serine-rich protein (PASrp) domain in *Polymycoviridae*, and the guanylyltransferase (Gtf) domain in *Spinareoviridae* [342,343,344]. Further details on the variation of genome organization among dsRNA mycovirus families can be found in Table 4.

**Table 4:** Genomic features and characteristics of dsRNA mycoviruses.

Families and Genera	Genome Size and Segmentation	Genome Organization	Exemplar Species
Family *Amalgaviridae* Mycoviral associated genera *Zybavirus*	Non-segmented genomes around 3.5 kb in size.	5′ proximal ORF encodes for a CP, followed by ribosomal frameshift signal and a 3′ proximal ORF encoding for an RdRp protein.	Zygosaccharomyces bailii virus Z
Family *Chrysoviridae* Mycoviral associated genera *Alphachrysovirus* *Betachrysovirus*	Multisegmented genomes around 8.9 to 16.0 kb in size. *Alphachrysovirus*: 3–4 separately encapsidated segments. *Betachrysovirus*: 4–7 separately encapsidated genomes.	dsRNA 1: Encodes for an RdRp. dsRNA 2: Encodes for a CP. dsRNA 3: Encodes for phytoreo S7 domain in *Alphachrysovirus*, and an unknown protein in *Betachrysovirus.* dsRNA 4–7: Encodes for a hypothetical protein with unknown function.	*Alphachrysovirus*: Penicillium chrysogenum virus *Betachrysovirus*: Botryosphaeria dothidea chrysovirus 1
Family *Megabirnaviridae* Mycoviral associated genera *Megabirnavirus*	Bisegmented genomes around 16 kb in size.	dsRNA 1: 5′ proximal ORF encodes for a CP, followed by a ribosomal frameshift signal and a 3′ proximal ORF encoding for an RdRp. dsRNA 2: Encodes for hypothetical protein with unknown function.	Rosellinia necatrix Megabirnavirus 1
Family *Partitiviridae* Mycoviral associated genera *Alphapartitivirus* *Betapartitivirus* *Gammapartivirus*	Bisegmented genomes around 3 to 4.8 kb in size.	dsRNA 1: Encodes for an RdRp. dsRNA 2: Encodes for a CP.	*Alphapartitivirus*: Rosellinia necatrix partitivirus 2 *Betapartitivirus*: Ceratocystis resinifera virus 1 *Gammapartivirus*: Aspergillus ochraceous virus
Family *Polymycoviridae* Mycoviral associated genera *Polymycovirus*	Multisegmented genomes around 7.5 to 12.5 kb in size.	dsRNA 1: Encodes for an RdRp. dsRNA 2: Encodes for hypothetical protein with unknown function. dsRNA 3: Encodes for a Mtf. dsRNA 4: Encodes for a PASrp. dsRNA 5–8: Encodes for hypothetical proteins with unknown functions.	Aspergillus fumigatus tetramycovirus 1
Family *Polymycoviridae* Mycoviral associated genera *Polymycovirus*	Multisegmented genomes around 7.5 to 12.5 kb in size.	dsRNA 1: Encodes for an RdRp. dsRNA 2: Encodes for hypothetical protein with unknown function. dsRNA 3: Encodes for a Mtf. dsRNA 4: Encodes for a PASrp. dsRNA 5–8: Encodes for hypothetical proteins with unknown functions.	Aspergillus fumigatus tetramycovirus 1
Family *Quadriviridae* Mycoviral associated genera *Quadrivirus*	Quadripartite genomes around 3.5 to 5.0 kb in size.	dsRNA 1: Encodes for a hypothetical protein with unknown function. dsRNA 2 and 4: Encode for a CP. dsRNA 3: Encodes for an RdRp.	Rosellinia necatrix quadrivirus 1
Family *Spinareoviridae * Mycoviral associated genera *Mycoreovirus*	Multisegmented genomes (11–12 segments) around 23 to 29 kb in size	Segments encodes for a single viral protein (VP1 to VP12) VP 1: Encodes for an RdRp VP 2: Encodes for CP VP 3 or VP10: Encodes for a capping enzyme, (guanylyltransferase) VP4 to VP12: Encodes for hypothetical proteins with unknown function	Mycoreovirus-1/Cp9B21
Family *Totiviridae * Mycoviral associated genera *Totivirus* *Victorivirus*	Non-segmented genomes around 4.6–7.0 kb in size	5′ proximal ORF encodes for CP and the 3′ proximal ORF encodes for an RdRp Totiviruses additionally encode for a ribosomal frameshift signal between the CP and RdRp Some totiviruses may additionally produce satellite dsRNA which encode for killer proteins	*Totivirus*: Saccharomyces cerevisiae virus L-A *Victorivirus*: Helminthosporium victoriae virus 190S
Family Unclassified Mycoviral associated genera *Botybirnavirus*	Bisegmented genomes around 5.7 to 6.3 kb in size	dsRNA 1: Encodes for an RdRp dsRNA 2: Encodes for a hypothetical protein with unknown function	Bipolaris maydis botybirnavirus 1

Compiled using data from the International Committee on Taxonomy of Viruses (ICTV): https://ictv.global/taxonomy/ (accessed on 20 March 2023), Nibert, Ghabrial, Maiss, Lesker, Vainio, Jiang and Suzuki [33], Krupovic, et al. [337], Ghabrial, Castón, Jiang, Nibert and Suzuki [5], Lin, et al. [345], and Li, et al. [346].

#### 3.2.2. Single Stranded RNA (ssRNA) Mycoviruses

##### Positive (+) Sense

Mycoviruses belonging to families with positive-sense single-stranded RNA (+ssRNA) genomes display considerable diversity in terms of genome size, with genomes ranging from approximately 2.0 kb to 17.6 kb [172,347]. The majority of +ssRNA mycoviruses have non-segmented genomes, however, the recently classified family *Hadakaviridae* consists of members with 10 to 11 genome segments [7]. While most mycoviruses replicate within the cytoplasm of their host, members of the *Mitoviridae* family demonstrate a unique replication pattern, taking place in the mitochondria of its fungal host [172]. More detail on the diversity of genome length and segmentation among +ssRNA mycovirus families and genera is presented in Table 5.

The presence of an RdRp domain is a characteristic feature of all +ssRNA mycoviruses. The genome of these viruses can include different protein domains, such as viral helicases (Hel), methyl transferases (Mtf), glycosyl transferases (Gtf), capsular polysaccharide synthases (CPS), phytoreo S7 domains, capsid proteins (CP), and proteases [347,348,349,350,351]. The location of the RdRp domain within the genome can vary among different mycovirus families, as can the specific protein domains present in their genomes. Further information on the variation of genome organization among +ssRNA mycovirus families can be obtained from Table 5.

##### Negative (−) Sense

Negative sense ssRNA mycoviruses belong to a single family, namely *Mymonaviridae*, which is characterized by filamentous, enveloped viruses with linear genomes that are around 6 to 10 kbp [352]. The genomes of −ssRNA viruses encode for one or more proteins, two of which are known, namely an RdRp protein and a nucleoprotein (NP) which encloses the viral genome [230,279,352,353]. The functions of all other ORF encoded proteins are still uncertain [352]. Further details on genome organization and genera within *Mymonaviridae* is given in Table 6.

##### Reverse Transcribing (RT)

Concerning reverse transcribing ssRNA mycoviruses, two families are currently recognized: *Metaviridae* and *Pseudoviridae*. Mycoviruses from the *Metaviridae* family are characterized by genomes ranging in length from 3 to 15 kb, while members of the *Pseudoviridae* family are generally shorter, ranging from 4 to 9 kb in length [354,355]. The replication process of these viruses involves reverse transcription within intracellular virus-like particles (VLP) to generate complementary DNA (cDNA), which is then integrated into the host chromosome through the action of an integrase protein [354,355].

The genome of RT ssRNA mycoviruses typically encodes for capsid (CP) or nucleocapsid proteins (NC), which are located on the gag gene, as well as protease (PR), reverse transcriptase (RT), integrase (INT), and ribonuclease H domains (RH), which are located on the pol gene [354,355,356]. The key difference between the two families lies in the fact that members of the Pseudoviridae family may encode the gag and pol proteins on separate open reading frames (ORFs), while members of the *Metaviridae* family encode these genes on a single ORF [354,355]. Further information on the different genera and characteristics of RT ssRNA viral families can be found in Table 7.

#### 3.2.3. Single-Stranded DNA (ssDNA) Mycoviruses

Mycoviruses with DNA genomes are rare compared to RNA viruses. There is currently only one recognized family containing ssDNA mycoviruses, namely *Genomoviridae*. This family encompasses two genera: *Gemycircularvirus* (represented by Sclerotinia sclerotiorum hypovirulence-associated DNA virus 1, SsHADV-1) and *Gemytripvirus* (represented by Fusarium graminearum gemytripvirus 1, FgGMTV1) [12,175]. These mycoviruses exhibit small genome sizes, which range from 1.3 kb to 2.4 kb [14,175]. The genomes of SsHADV-1 and FgGMTV1 encode for a replicase protein (REP) and a capsid protein (CP), with the former encoded on a single circular ssDNA genome, and the latter encoded on three circular ssDNA components (DNA-A, DNA-B, and DNA-C). ssDNA mycoviruses are not exclusively found in *Genomoviridae*, however. For example, geminivirus-like ssDNA has also been discovered in *Macrophomina phaseolina* (M. phaseolina DNA virus; MpDV) and *Mucor racemosus* (M. racemosus DNA virus; MrDV) [53]. Additionally, a novel ssDNA mycovirus, Gigaspora circovirus A has also been discovered and belongs to the *Circoviridae* family but has not yet been assigned to the genus [52]. Unlike other circoviruses which encode for both a REP and CP, Gigaspora circovirus A encodes for a REP only [52]. Although not officially recognized by the ICTV, Gigaspora circovirus A might represent the first member of *Circoviridae* which infects a fungal host [14,175]. Table 8 summarizes the properties of ssDNA mycoviruses which are currently recognized by ICTV.

## 4. Effect of Mycoviruses on Fungi

By being obligate intracellular parasites mycoviruses depend on host genes and gene products for their replication, which will in turn affect how the host phenotype manifests [6]. The majority of mycoviruses described so far have had no observed effect on their fungal hosts, although some can alter their host’s phenotypic traits [5]. These mycoviruses may change the virulence of their fungal hosts, often by altering the transcriptome profiles of their fungal hosts by interfering with protein–protein interactions and silencing antiviral proteins [1,6]. It should be noted, however, that these associations are sometimes complex. Several environmental factors, including the growth media [158], temperature [357], other mycoviruses [358], and the fungal plant or animal host [186] may also play a role in the outcome of the fungal host phenotype.

### 4.1. Host–Virus Arms Race

Mycoviral evasion of host defenses and underlying mechanisms of antiviral mechanisms are commonly studied using *Cryphonectria parasitica*, the model organism for mycoviral research [21,359,360], and more recently *Neurospora crassa* [198].

*C. parasitica* can defend against viral infection by use of an RNA-mediated gene regulation mechanism known as RNA silencing or RNA interference (RNAi) which commonly involves the use of endoribonucleases known as Dicer-like proteins (DCLs), and also Argonaute-like proteins (AGLs) [361,362,363]. DCL and AGL proteins form part of the RNA-induced silencing complex (RISC), which recognizes and cleaves the dsRNA genomes, or replicative intermediates, of infecting mycoviruses [6]. An in-depth review of RNA silencing mechanisms is covered by Zhao and Guo [364].

Mycoviruses may also suppress RNA silencing. Through proteins such as p29 and p27, Cryphonectria hypovirus 1 (CHV-1) and Cryphonectria hypovirus 2 (CHV-2) suppress this pathway in *C. parasitica*, allowing enhanced replication and altered symptoms in the host [365,366]. Similarly, Rosellinia necatrix mycoreovirus 3 (RnMYRV-3) produces S10, which also helps suppress silencing in *Rosellinia necatrix* [367]. In *N. crassa*, RISC-associated proteins are upregulated after infection with Neurospora crassa fusarivurs 1 (NcFV1), but AGL proteins are downregulated post-transcriptionally [198]. On the other hand, some mycoviruses, such as Talaromyces marneffei partitivirus-1 (TmPV- 1), downregulate the mRNA levels of DCL and AGL proteins in the fungal host [110].

Interestingly, some types of fungi do not have an RNAi pathway, rendering them highly susceptible to mycovirus infection. For example, the persistence and replication of MsMV-1, a mycovirus of *Malassezia sympodialis* (a yeast linked to common skin disorders, pancreatic cancer, and Chron’s disease), is thought to be due to the absence of an RNAi pathway within this organism [311,368,369,370].

### 4.2. Hypervirulence, Hypovirulence and the Effect of Mycoviruses on the Pathogenesis of Fungi

Mycoviruses that induce hypervirulence increase the virulence of the fungal host [6]. Despite its perceived negative effects, hypervirulence may also have desirable traits in plant pathogenic fungi. For example, amycovirus known as Leptosphaeria biglobosa quadrivirus 1 (LbQV-1) induces hypervirulence in *Leptosphaeria biglobosa* [77]. The infected strains of *L. biglobosa* confer systemic resistance and protect the host plant, *Brassica napus* (oilseed rape or canola), from a closely related and more aggressive fungus called *Leptosphaeria maculans* [77]. Some mycoviruses also confer hypervirulence to entomopathogenic fungi from the genera *Metarhizium* and *Beauveria*, which are routinely used as biocontrol agents against a few arthropod pests [371]. In these circumstances, hypervirulence is a more desirable trait as it may increase the effectiveness of fungicides that are currently on the market.

Mycoviruses that induce hypovirulence, adversely affect the virulence, morphology, sporulation, growth rate, and pigmentation of their fungal hosts [6,15,16]. Although the precise nature and molecular pathways by which these viruses cause hypovirulence are still largely unclear, they are known to exert some of their effects through virus–host protein–protein interactions [6]. Mycovirus-induced hypovirulence of *C. parasitica* can be attributed to the alteration of numerous signal transduction pathways, including those important for virulence [372,373,374]. For example, the reduction of pigmentation, sporulation, and laccase accumulation in *C. parasitica* is linked to the CHV-1 papain-like protease p27 [375]. CHV-1 infected *C. parasitica* additionally upregulates ATG8, a homolog of ubiquitin-like yeast autophagy protein, since it is essential for replicating this virus [376]. In contrast, CHV-1 also differentially regulates proteins that prevent the expression of viral RNA, such as DNA methyltransferases, which in turn results in retarded growth and aberrant colony morphology of the host [377].

Mycoviruses that induce hypovirulence have been discovered in various plant pathogenic fungi, including several *Fusarium* spp. [349,378,379], the white root rot fungus *R. necatrix* [213,215,380], white mold fungus *S. sclerotiorum* [12,16,381], rice blast fungus *Magnaporthe oryzae* [382], and the grey mold rot fungi *Botrytis cinerea* [39,383,384] and *Alternaria* spp. [82,385]. Hypovirulence-inducing mycoviruses are of immense interest to plant pathologists, due to their potential use as biocontrol agents of plant pathogenic fungi.

The precise impact of mycoviruses on pathogenic fungi affecting human health remains largely unknown, yet a handful of studies have shed some light on these relationships. For example, infection with MsMV-1 appears to result in significant transcriptional rewiring in *M. sympodiali*, causing upregulation of transcriptional factors and ribosomal genes, while simultaneously repressing genes responsible for cellular metabolism [311]. Interestingly, the MsMV-1 putative mycoviral toxin also elicits an immune response in macrophages and augments the ability of infected isolates to colonize murine skin [311]. This suggests that the mycovirus may play a role in the pathogenicity of the host fungus. Similarly, recent investigations have discovered the presence of antibodies against a certain strain of mycovirus-infected *Aspergillus flavus* (MCAF) in the plasma of patients that were in remission from acute lymphoblastic leukemia (ALL) [386]. Within this study, the exposure of mononuclear blood cells from ALL patients in remission to the supernatant of a mycovirus-containing *Aspergillus flavus* resulted in a significant reappearance of cell surface and genetic markers consistent with this disease [386]. The authors, therefore, hypothesize that exposure to MCAF may contribute to the development of ALL [386]. These studies show that the role of mycoviruses in diseases caused by human pathogenic fungi, with and without their hosts, needs to be further explored.

### 4.3. The ‘Killer Phenotype’ in Yeasts

Mycoviruses do not exclusively infect filamentous fungi but have also been associated with various species of yeast [264,268]. Some induce the ‘yeast killer phenotype’, eliminating competing yeasts and providing a competitive advantage to the host [387]. The killer phenotype in the model organism *S. saccharomyces* is normally determined by two co-infecting totiviruses with a mutualistic relationship, namely the helper LA and satellite M virus [388]. ScV (Saccharomyces Cerevisiae virus) -L-A encodes essential proteins for replication and transcription, while satellite M encodes for a preprotoxin which provides immunity to the host and kills off non-infected cells when processed into toxin [387]. The toxins generated by this satellite virus vary among strains and can induce cell death through different mechanisms. These include the disruption of cytoplasmic membrane function and the formation of lethal ion channels (K1 and K2), as well as cell cycle arrest in the G1 or early S phase (K28) [389,390,391].

Industrial yeast strains with virally encoded killer systems are highly sought after for their ability to restrain spoilage microorganisms and preserve the quality of food products and beverages [392,393,394]. For example, *Ustilago maydis*, *Kluyveromyces wickerhamii*, *Pichia anomala*, and *Pichia membranifaciens* produce virally encoded toxins that have antifungal activity against the wine spoilage yeast, *Brettanomyces bruxellensis* [395,396]. They have also been discovered in *Zygosaccharomyces bailii*, where they encode for the toxin zygocin, which has broad antifungal activity and has the potential as an antimycotic drug [397].

## 5. Mycoviruses as Biocontrol Agents

Among the best-known applications of a mycovirus in the field is CHV-1 against the chestnut blight pathogen, *C. parasitica* [398,399]. Under typical conditions, *C. parasitica* infections result in the appearance of cankers on the stems and branches of susceptible trees, which destroy the cambium tissue and ultimately result in tree death [400]. CHV-1 infected strains, on the other hand, have reduced virulence, resulting in superficial cankers which eventually stop growing and become passive [400]. Mycovirus-based biocontrol of chestnut blight has proven to be a great success in Europe, largely due to the low genetic diversity among fungal strains [400]. In regions where naturally hypovirulent strains of *C. parasitica* were present, CHV-1 effectively spread without intervention [401]. However, in areas with little to no natural hypovirulence, CHV-1 could also be artificially introduced by treating bark cankers with hypovirulent *C. parasitica* [402,403]. Research into hypovirulence-associated mycoviruses in other plant pathogenic fungi has since been prompted by the success of hypovirus-mediated hypovirulence in *C. parasitica*.

The Sclerotinia sclerotiorum hypovirulence-associated DNA virus 1 (SsHADV1) is infectious as purified particles, and can directly infect the hyphae of *S. sclerotiorum* [404]. Researchers have thus developed an aerial spray capable of killing *S. sclerotiorum* on infected rapeseed plants in the field, by using hyphal fragments from an infected strain of the fungus [404]. Sclerotinia sclerotiorum partitivirus 1 (SsPV1), another mycovirus of *S. sclerotiorum*, appears to spread through hyphal contact to different strains regardless of vegetative incompatibility [20]. This suggests that SsPV1 also has potential as a biocontrol agent against *S. sclerotiorum* in the field [20].

While the focus of mycoviral research has predominantly centered around edible mushrooms and plant pathogenic fungi, an emerging area of investigation pertains to the search for mycoviruses with potential therapeutic benefits for human health [107,357,405].

### 5.1. Limitations of Biocontrol: The Role of Mycovirus Transmission

Despite their immense potential, the use of mycoviruses as biocontrol agents of plant pathogenic fungi is complicated by their mode of transmission. Mycoviruses lack an extracellular route of transmission [3]. Instead, they can only spread intracellularly, through hyphal anastomosis (horizontal transmission) or sporulation (vertical transmission) [5].

#### 5.1.1. Horizontal Transmission

One of the greatest barriers to the successful spread of mycoviruses pertains to hyphal anastomosis. Hyphal anastomosis occurs when specialized hyphae from the same fungus, or hyphae from different fungi fuse and exchange cytoplasmic content, which includes any associated mycoviruses [406]. However, in order for the hyphae from different fungal strains or species to fuse, they need to be vegetatively compatible [3]. Vegetative compatibility is determined by the fungal vegetative incompatibility genes (*vic* genes), which will trigger programmed cell death (PCD) when contact between incompatible fungi occurs [407]. Thus, mycoviruses cannot be transmitted from a hypovirulent fungal strain to a target fungal strain if they are vegetatively incompatible [21]. For example, CHV1 has been effective against *C. parasitica* in Europe, but not in America, where there is greater diversity of *vic* groups between fungal strains [401,408]. Similarly, although *S. sclerotiorum* harbours a diverse range of mycoviruses, their use in the field is restricted due to the high diversity of *vic* loci among different strains which may also be very complicated under field conditions [409,410]. Research is underway to resolve the issue of vegetative incompatibility as it relates to mycovirus transmission.

Many methods and techniques have been developed in order to study vegetatively compatibility systems in fungi and to find ways to overcome the *vic* system. For example, *vic* genes related to five to six loci in *C. parasitica* have been linked to vegetative incompatibility and virus transmission in one study [21]. The disruption of these genes then allowed for the development of a super donor strain, which allowed the spread of mycoviruses between incompatible strains [411]. This approach will not be feasible for all fungi, however, as some plant pathogenic fungi may have more complicated *vic* systems that result in a high *vic* diversity [16,228]. Chemical compounds have been used to enhance viral transmission and to prevent programmed cell death (PCD), which is caused by vegetative incompatibility between fungi [412]. When vegetatively incompatible strains of *R. necatrix* were cultured together on a medium supplemented with zinc, hyphal anastomosis improved and mycoviruses could be transmitted to isolates of different VCGs [413].

While vegetative incompatibility prevents transmission of mycoviruses in most cases, other factors also often play a role. In situ inoculation on chestnut wood increased transmission efficiency of CHV1 between vegetatively incompatible strains [414]. In another study, two vegetatively incompatible strains of *R. necatrix*, one of which contained a mycovirus, were inoculated on apple trees and later found to harbor the same mycovirus [415]. This may be due to several factors, including different environmental conditions, and a weakened vegetative incompatibility response due to environmental microorganisms or the host plant itself [407]. Research has also indicated that horizontal transmission of mycoviruses between fungal and plant hosts may occur in cases of co-infection with plant viruses [40]. In such instances, the replication of these viruses in both hosts is facilitated. This phenomenon has been linked to suppressing antiviral mechanisms in both the plant and fungus [40]. For example, the mycovirus CHV-1 produces a protein called p29 that downregulates components of the antiviral RNAi system in fungal hosts, thereby promoting virus accumulation [365]. However, research by Bian et al. [39] has shown that this protein has limited functionality in certain plant hosts, such as *N. tabacum*. TMV on the other hand, encodes for a replicase that interferes with the antiviral response in the plant host, thereby enhancing CHV-1 accumulation in the plant [40]. Additionally, TMV produces a cell-to-cell movement protein (MP) that is typically absent in mycoviruses, thus enabling the dissemination of CHV-1 throughout the *N. tabacum* [365]. This may then enhance the ability of the mycovirus to access other fungi that may have established themselves in the same plant [40]. Conversely, CHV-1 inhibits the fungal antiviral defense mechanism, which typically acts to eliminate the plant virus [40]. This, in turn, permits the accumulation of TMV within the fungal host, specifically *F. graminearum*, as demonstrated in this study [40].

Different viral strains also play a role in transmission efficiency, where researchers have found that strains with higher virulence have higher transmissibility [416].

Interestingly, several mycoviruses are known to either influence the host *vic* system to transmit between vegetatively incompatible strains or infect the host directly as infectious particles [188,234,404,417]. CHV-1 for example, has been found to downregulate genes that are involved in programmed cell death (PCD), which occurs after vegetatively incompatible strains interact, thus allowing for transmission between incompatible strains [417,418]. Co-infection of mycoviruses can also result in the transmission of viruses between vegetatively incompatible fungi. One study has demonstrated that Sclerotinia sclerotiorum mycoreovirus 4 (SsMYR4) downregulates cellular activities and pathways associated with vegetative incompatibility mediated PCD [419]. This in turn facilitated the horizontal transmission of other hypovirulent co-infecting viruses [419]. Mycoviruses with these traits hold immense potential as biocontrol agents; however, more research is required to fully understand the mechanisms behind these phenomena.

Regarding their clinical application, mycoviruses that induce hypovirulence would require administration through delivery methods such as injection or topical application to the target fungus of an infected patient [107]. The lack of extracellular replication in mycoviruses is caused by the impenetrable fungal cell wall acting as a barrier against mycovirus uptake, thus limiting their potential as therapeutic agents against human pathogenic fungi [420]. Hyphal anastomosis is a possible alternative, as noted previously, various challenges preclude its practical use in human patients [107]. A promising avenue for the development of therapeutic interventions for humans has emerged with the discovery of ssDNA mycoviruses that are capable of extracellular transmission in *S. sclerotiorum* [107,404]. It is thus plausible that ssDNA viruses may represent the most viable candidates for therapeutic applications in humans. Because mycoviruses lack an extracellular route of infection, transfection, or transformation using full-length viral cDNA clones, purified virus particles, and in vitro RNA transcripts are usually used to transmit mycoviruses between incompatible fungi in the laboratory [421,422,423]. The development of such clones is complex, however, especially in the case of multisegmented mycoviruses [407]. Nevertheless, studies have demonstrated that encapsulated mycoviruses can be transformed into fungal protoplasts using polyethylene glycol-mediated protocols, which is now the standard approach for transmission in a laboratory setting [12,424,425]. Mycoviruses can also be transmitted between incompatible fungal strains through protoplast fusion, which has the advantage of allowing transmission of both encapsulated and unencapsulated viruses [22]. Although these methods normally study host and viral factors involved in viral replication or symptom induction, they have also been used to expand the host range for some mycoviruses [22].

Vectors, such as insects or parasites that transmit mycoviruses between fungi, may be used to overcome barriers of vegetative compatibility and rapidly help mycoviruses establish populations in the field [20]. For example, the transmission of SsHADV-1 by frugivorous insects to other vegetatively incompatible strains has been observed under laboratory conditions, for example [20]. However, producing and dispersing such vectors in the field is impractical [20]. Hence, it is more feasible to exploit a naturally occurring vector, such as mycoparasites, which can transmit the mycovirus via hyphal parasitization [20]. A hypovirulence-associated mycovirus can thus be introduced into the mycoparasite using transfection techniques or dual culturing and then used to infect a host fungus [20].

Most mycoviral research regarding biocontrol focuses on hypovirulence-inducing viruses, but some researchers have also exploited the “killer phenotypes” of dimorphic fungi to confer resistance to plant hosts. For example, one study has shown that transgenic expression of the viral KP4 killer toxin from *U. maydis* into Swiss wheat confers resistance to these crops against *U. maydis* and related hosts [426]. These killer systems are not generally present in filamentous fungi, but similar strategies could also be explored for plant pathogenic yeasts or dimorphic fungi.

#### 5.1.2. Vertical Transmission

For mycoviruses to be considered effective biocontrol agents, they must not only exhibit hypovirulence and have the ability to transmit to uninfected fungi through hyphal anastomosis, but they must also demonstrate efficient transmission to the fungal progeny. This requirement is crucial for the long-term efficacy of mycovirus-based biocontrol strategies. The transmission of some mycoviruses to the fungal progeny occurs primarily through sporulation, which can be sexual or asexual [1,3,15]. Transmission rates, however, vary greatly between fungus-virus combinations and between different spore types (asexual vs. sexual) [15]. For example, one study has demonstrated that the transmission of CHV1, 2, 3, and 4 to the ascospore progeny of *C. parasitica* is ineffective as infection results in a loss of female fertility [118]. In contrast to this, other studies have shown that mycoviruses can be transmitted to the ascospore progeny of *C. parasitica* strains which are infected by reoviruses (Mycoreovirus 1 and 2) or a mitovirus (Cryphonectria mitovirus-1) [118,427]. Vertical transmission through asexual spores or other asexual structures such as sclerotia are commonly observed in mycoviruses [228,379,428,429]. Disseminating mycoviruses into asexual spores allows the spread of these viruses within their host to longer distances than permitted by horizontal transmission alone and allows mycoviruses to persist within the fungal population [414]. Mycovirus-based biocontrol strategies may face challenges with vertical transmission through asexual fragments. In some cases, hypovirulence has been observed to be linked with the disruption of conidia, resulting in lower transmission rates. For instance, CHV-2 induces substantial hypovirulence in *C. parasitica*, but has limited transmission to conidia with only a 2–5% transmission rate, leading to its restricted geographical distribution [430]. It is important to note that this scenario may not be universal. According to a study by Lee, et al. [431], four mycoviruses, which reduce the virulence of *Fusarium graminearum*, exhibit more efficient transmission to conidia than those that cause symptomless infections. Another issue related to the transmission of fungal pathogens through asexual spores is that some fungal species either do not produce conidia or the conidia play a minimal role in their life cycle [407].

In general, the transmission of mycoviruses to sexual spores (ascospores, basidiospores, etc.) is believed to be less prevalent than the transmission of mycoviruses to asexual spores (conidia) [20]. However, studies now indicate that this transmission mode may be more common than previously thought [118,232,432,433]. The mechanisms behind mycovirus transmission via sexual spores remain unclear, but it is believed that this could represent a potential pathway for exchanging mycoviruses among different *vic* groups [407]. Hence, an increased focus on investigating these mechanisms could provide new avenues for controlling mycovirus transmission and boosting the potential of biological control efforts.

## 6. The Detection of Mycoviruses

### 6.1. In Vitro Based Detection

In the past, mycoviruses were primarily detected by the use of a culture-based approach. The basis for culture-based detection is rooted in the observation that most mycoviruses have dsRNA genomes, or a dsRNA replicative intermediate, which are not generated by the host [5,15]. The standard method used to detect mycoviruses with RNA genomes involves purifying dsRNA from total fungal RNA extracts, by using cellulose chromatography or other column-based approaches [434,435]. While these approaches are usually rapid and inexpensive, they suffer from several disadvantages [2]. Using an in vitro-based approach may lead to overestimation of dsRNA or ssRNA levels since dsRNA enrichment protocols are commonly used [3]. Moreover, these techniques strengthen the idea that mycovirus genomes are primarily composed of RNA and completely disregard viruses that may be composed of DNA [2]. Purified dsRNA is also normally visualized using agarose gel electrophoresis, which may result in false negatives in cases where there are only low-titer infections.

Culture-based methods are also used to evaluate the effect of mycoviral infection on the fungal host. Infected cultures may show decreased growth and sporulation, as well as alteration in pigmentation and morphology [6]. For example, CHV-1-infected *C. parasitica* are deficient in the characteristic orange pigmentation of non-infected strains. In contrast, infected *S. sclerotiorum* strains show abnormal colony morphology and show smaller and fewer sclerotia [6]. However, this is not always the case. Some mycoviruses that induce hypovirulence in vitro, may induce hypervirulence in planta [84]. Consequently, dsRNA profiling is usually used alongside in silico methods to identify and characterize mycoviruses [2,435].

### 6.2. In Silico Based Detection

Thanks to the development of new technologies, it has become easier to detect mycoviruses and gain insight into their structure and biology [2,20,436]. By interrogating the metatranscriptomic datasets of fungal hosts for sequences showing homology to mycoviral proteins, the in silico approach can identify any associated putative mycoviruses. One such protein is the RdRp, which is essential for viral transcription and replication, and is thus ubiquitous in RNA viruses [2]. Other proteins which are also found in the genomes of some, but not all mycoviruses, are viral helicases, glycosyl transferases, methyl transferases, and capsid proteins [211,437,438]. Because the in silico approach to detection does not make use of agarose gel electrophoresis, it is more sensitive to low titer mycoviral infections than in vitro-based approaches [2]. The greater accessibility of fungal transcriptomic datasets on open-access platforms like the NCBI sequencing reads archive (SRA), as a result of decreased sequencing costs, is another benefit of in-silico-based approaches [61]. Researchers can now examine publicly available fungal datasets for the presence of these viruses, further elucidating the diversity and prevalence of mycoviruses in under-researched fungal groups.

Some studies now use approaches such as genome-wide linkage analysis to understand the underlying mechanisms behind the effects that mycoviruses have on their fungal hosts [431]. For instance, RNA-seq-based genome-wide expression analyses showed distinct expression patterns in response to infection by four phylogenetically different mycoviruses in *F. graminearum* (FgV1-4) [431]. Even though these mycoviruses all showed changes in transcriptome expression, only FgV1 and FgV2 caused observable changes in the host phenotype [431]. Mycoviruses are dependent on many host factors, as well as pathways and processes related to metabolism, transport, RNA processing, and signaling, and not all of these will result in a phenotypic change in the host [3]. Mycoviruses and their hosts often interact in complex ways, so more detailed research is needed to better understand these interactions.

## 7. Conclusions

Mycoviruses are ubiquitous within the fungal kingdom. Recent studies have shown that they are associated with most of the major fungal taxa. Yet, the number of mycoviruses that have been fully characterized is low in comparison to economically important plant and animal viruses. This is most likely due to their cryptic nature, which means that it is often difficult to distinguish whether a fungal host is infected by empirical observation. Mycoviruses have been commonly detected with in vitro-based approaches, however, these are known to suffer from several disadvantages. The rapid advances in high throughput sequencing however, particularly RNA sequencing, which has become mainstream, has led to an exponential increase in the number of mycoviruses that have been discovered. Fungal transcriptomes can now be mined for mycoviral sequences, and this allows for the study of the complex interactions between fungal hosts and mycoviruses. Unlike most other viruses, mycoviruses do not always elicit measurable changes to host phenotypes. It is now clear that some mycoviruses cause hypovirulence and may be potential biocontrol agents of plant pathogenic fungi, which makes their discovery and characterization in such hosts even more important. There are, however, still numerous challenges that need to be addressed before their widespread use. Research is ongoing in pursuing the use of mycoviruses as biocontrol agents, and it is conceivable that these challenges will be overcome in the future.

## Figures and Tables

**Table 1:** Mycoviruses associated with Ascomycota.

Ascomycota				
Class	Order	Family	Genera	Mycoviruses
Arthoniomycetes	Arthoniales	*Chrysotrichaceae*	*Chrysothrix*	dsRNA [42]
Dothideomycetes	Botryosphaeriales	*Aplosporellaceae*	*Aplosporella*	dsRNA [43]
		*Botryosphaeriaceae*	*Botryosphaeria*	dsRNA and +ssRNA [44,45,46,47,48]
			*Diplodia*	dsRNA and +ssRNA [49,50,51]
			*Eutiarosporella*	+ssRNA [52]
			*Macrophomina*	dsRNA, +ssRNA, −ssRNA and ssDNA [53,54,55,56]
			*Neofusicoccum*	dsRNA and +ssRNA [57,58,59]
			*Phaeobotryon*	dsRNA [60]
		*Phyllostictaceae*	*Phyllosticta*	dsRNA [61]
	Capnodiales	*Cladosporiaceae*	*Cladosporium*	+ssRNA, −ssRNA and RT ssRNA [62,63]
		*Mycosphaerellaceae*	*Cercospora*	+ssRNA and −ssRNA [64]
			*Dothistroma*	dsRNA [65]
			*Mycosphaerella*	+ssRNA [52,66]
			*Pseudocercospora*	dsRNA [66]
			*Zymoseptoria*	dsRNA and +ssRNA [61,64,67]
	Dothideales	*Saccotheciaceae*	*Aureobasidium*	+ssRNA [52]
	Mycosphaerellales	*Teratosphaeriaceae*	*Acidomyces*	+ssRNA [61]
			*Hortaea*	dsRNA [61]
	Pleosporales	*Coniothyriaceae*	*Coniothyrium*	dsRNA and −ssRNA [63,68]
		*Corynesporascaceae*	*Corynespora*	dsRNA [67,69]
		*Cucurbitariaceae*	*Cucurbitaria*	dsRNA [69,70]
		*Delitschiaceae*	*Delitschia*	dsRNA [61]
		*Didymellaceae*	*Didymella*	dsRNA [71]
			*Epicoccum*	+ssRNA [63]
			*Leptosphaerulina*	+ssRNA [63]
			*Phoma*	dsRNA and +ssRNA [71,72,73,74,75]
			*Stagonosporopsis*	dsRNA [47,72,73,74,75]
		*Leptosphaeriaceae*	*Leptosphaeria*	dsRNA and + ssRNA [76,77,78]
		*Lindgomycetaceae*	*Clohesyomyces*	dsRNA [47,61]
		*Massarinaceae*	*Helminthosporium*	dsRNA [79]
		*Periconiaceae*	*Periconia*	dsRNA and +ssRNA [61,80]
		*Pleosporaceae*	*Alternaria*	dsRNA, +ssRNA, and −ssRNA [63,80,81,82,83,84,85,86,87,88]
			*Bipolaris*	dsRNA and +ssRNA [89,90,91,92]
			*Cochliobolus*	dsRNA [93]
			*Curvularia*	dsRNA [94]
			*Drechslera*	dsRNA [61,95]
			*Exserohilum*	dsRNA [95,96,97]
			*Pleospora*	dsRNA [96,97,98]
			*Setosphaeria*	dsRNA and +ssRNA [97,99,100]
			*Stemphylium*	+ssRNA [101]
Eurotiomycetes	Chaetothyriales	*Herpotrichiellaceae*	*Phialophora*	dsRNA [81,82,83,84,85,86,87,88,102,103]
	Eurotiales	*Aspergillaceae*	*Aspergillus*	dsRNA and +ssRNA [93,104,105,106,107]
			*Penicillium*	dsRNA, +ssRNA and −ssRNA [15,63,89,90,91,92,108,109]
		*Trichocomaceae*	*Talaromyces*	dsRNA [94,110,111,112]
	Onygenales	*Ajellomycetaceae*	*Blastomyces*	dsRNA [98,113]
		*Ascosphaeraceae*	*Ascosphaera*	+ssRNA [97,99,100,114,115]
		Onygenales incertae sedis	*Myriodontium*	dsRNA [116]
Lecanoromycetes	Lecanorales	*Stereocaulaceae*	*Lepraria*	dsRNA [42,101]
Sordariomycetes	Diaporthales	*Cryphonectriaceae*	*Cryphonectria*	dsRNA, +ssRNA and −ssRNA [117,118,119,120,121]
			*Endothia*	dsRNA [122]
		*Diaporthaceae*	*Diaporthe*	dsRNA and +ssRNA [56,123,124]
		*Melanconiellaceae*	*Melanconiella*	+ssRNA [125]
		*Valsaceae*	*Cytospora*	dsRNA [126]
			*Phomopsis*	dsRNA and +ssRNA [127,128,129,130]
			*Valsa*	+ssRNA [131]
	Glomerellales	*Glomerellaceae*	*Colletotrichum*	dsRNA and +ssRNA [132,133,134,135,136,137,138]
		*Plectosphaerellaceae*	*Sodiomyces*	dsRNA and +ssRNA [139]
			*Verticillium*	dsRNA and +ssRNA [140,141,142,143]
	Hypocreales	*Bionectriaceae*	*Clonostachys*	dsRNA [144]
		*Clavicipitaceae*	*Atkinsonella*	dsRNA [145]
			*Epichloë*	dsRNA [146,147]
			*Metarhizium*	dsRNA [148,149,150,151]
			*Ustilaginoidea*	dsRNA [152,153,154,155,156]
		*Cordycipitaceae*	*Beauveria*	dsRNA [61,157,158,159,160]
			*Cordyceps*	dsRNA [161,162]
			*Isaria*	dsRNA [163]
		*Hypocreaceae*	*Hypomyces*	dsRNA [164]
			*Trichoderma*	dsRNA and +ssRNA [61,165,166,167,168]
			*Fusarium*	dsRNA, +ssRNA, −ssRNA, and ssDNA [169,170,171,172,173,174,175]
			*Ilyonectria*	dsRNA [176,177]
			*Nectria*	dsRNA [178]
			*Rugonectria*	dsRNA and +ssRNA [179]
			*Thelonectria*	dsRNA [180]
		*Hypocreales incertae sedis*	*Acremonium*	+ssRNA [63]
		*Ophiocordycipitaceae*	*Ophiocordyceps*	+ssRNA [61]
			*Tolypocladium*	dsRNA [181,182]
	Magnaporthales	*Magnaporthaceae*	*Buergenerula*	+ssRNA [183]
			*Gaeumannomyces*	dsRNA and +ssRNA [61,184]
			*Magnaporthe*	dsRNA and +ssRNA [185,186,187,188]
	Microascales	*Ceratocystidaceae*	*Berkeleyomyces*	dsRNA and +ssRNA [189,190]
			*Ceratocystis*	dsRNA and +ssRNA [191]
			*Endoconidiophora*	dsRNA [192]
		*Pyriculariaceae*	*Pyriculariaceae*	dsRNA [193,194]
	Ophiostomatales	*Ophiostomataceae*	*Grosmannia*	dsRNA and +ssRNA [61]
			*Ophiostoma*	dsRNA and +ssRNA [195,196,197]
	Sordariales	*Sordariaceae*	*Neurospora*	dsRNA and +ssRNA [198]
	Togniniales	*Togniniaceae*	*Phaeoacremonium*	dsRNA and +ssRNA [63]
	Xylariales	*Apiosporaceae*	*Nigrospora*	dsRNA and +ssRNA [199,200,201,202]
		*Diatrypaceae*	*Monosporascus*	dsRNA [203]
		*Hypoxylaceae*	*Annulohypoxylon*	+ssRNA [204]
		*Microdochiaceae*	*Microdochium*	+ssRNA [205]
		*Sporocadaceae*	*Pestalotiopsis*	dsRNA, +ssRNA and −ssRNA [206]
			*Pseudopestalotiopsis*	dsRNA [207]
		*Xylariaceae*	*Entoleuca*	dsRNA and +ssRNA [208,209,210]
			*Rosellinia*	dsRNA and +ssRNA [18,208,211,212,213,214,215,216,217]
Leotiomycetes	Leotiomycetes incertae sedis	*Myxotrichaceae*	*Oidiodendron*	+ssRNA [70,218]
		*Pseudeurotiaceae*	*Pseudogymnoascus*	dsRNA [219]
	Erysiphales	*Erysiphaceae*	*Erysiphe*	dsRNA and +ssRNA [68,220,221]
			*Podosphaera*	dsRNA and +ssRNA [77]
	Helotiales	*Godroniaceae*	*Gremmeniella*	dsRNA and +ssRNA [51,61,222,223]
		*Helotiaceae*	*Hymenoscyphus*	dsRNA, +ssRNA and −ssRNA [121,224,225]
		*Mollisiaceae*	*Loramyces*	+ssRNA [61]
		*Rutstroemiaceae*	*Rutstroemia*	+ssRNA [61]
		*Sclerotiniaceae*	*Botrytis*	dsRNA and +ssRNA [226,227,228,229,230,231]
			*Monilinia*	dsRNA, +ssRNA and ssDNA [232,233]
			*Sclerotinia*	dsRNA, +ssRNA, −ssRNA and dsRNA [16,175,234,235,236,237,238]
			*Sclerotium*	dsRNA and +ssRNA [239,240]
	Thelebolales	*Thelebolaceae*	*Thelebolus*	dsRNA [61]
Pezizomycetes	Pezizales	*Caloscyphaceae*	*Caloscypha*	dsRNA [241]
		*Discinaceae*	*Gyromitra*	dsRNA [61,242]
		*Morchellaceae*	*Morchella*	dsRNA and +ssRNA [61,243]
		*Pezizaceae*	*Peziza*	+ssRNA [204]
			*Sarcosphaera*	dsRNA [244]
			*Terfezia*	dsRNA [245]
		*Pyronemataceae*	*Geopora*	dsRNA and +ssRNA [121,246]
			*Picoa*	dsRNA and +ssRNA [247]
		*Tuberaceae*	*Tuber*	dsRNA, +ssRNA and RT-ssRNA [121,248,249,250]
Saccharomycetes	Saccharomycetales	*Debaryomycetaceae*	*Scheffersomyces*	dsRNA [251]
			*Wickerhamia*	dsRNA [252]
		*Dipodascaceae*	*Geotrichum*	dsRNA [253,254]
			*Magnusiomyces*	dsRNA [255]
			*Yarrowia*	dsRNA [256]
		*Phaffomycetaceae*	*Wickerhamomyces*	dsRNA [257]
		*Pichiaceae*	*Pichia*	dsRNA [258]
		*Saccharomycetaceae*	*Candida*	RT-ssRNA [259]
			*Saccharomyces*	dsRNA, +ssRNA and RT-ssRNA [260,261,262,263]
			*Torulaspora*	dsRNA [257]
			*Zygosaccharomyces*	dsRNA [264,265]
		*Saccharomycodaceae*	*Hanseniaspora*	dsRNA [264,266]
		*Saccharomycetales incertae sedis*	*Ambrosiozyma*	dsRNA [258]
			*Nadsonia*	dsRNA [267]
			*Starmerella*	dsRNA [268]
			*Magnaporthe*	dsRNA and +ssRNA [185,186,187,188]

**Table 2:** Mycoviruses associated with Basidiomycota.

Basidiomycota				
Class	Order	Family	Genera	Mycoviruses
Agaricomycetes	Agaricales	*Agaricaceae*	*Agaricus*	dsRNA and +ssRNA [269,270,271]
			*Leucocoprinus*	+ssRNA [52]
		*Clitocybaceae*	*Clitocybe*	+ssRNA [272]
		*Cyphellaceae*	*Chondrostereum*	dsRNA [204,273]
		*Hydnangiaceae*	*Laccaria*	+ssRNA [204]
		*Hygrophoraceae*	*Hygrophorus*	dsRNA [121,274,275]
		*Hymenogastraceae*	*Hebeloma*	dsRNA [276]
		*Lyophyllaceae*	*Leucocybe*	dsRNA [277]
		*Marasmiaceae*	*Moniliophthora*	+ssRNA [278]
		*Nidulariaceae*	*Cyathus*	+ssRNA [52]
		*Omphalotaceae*	*Collybiopsis*	+ssRNA [204]
			*Lentinula*	dsRNA and +ssRNA [204]
		*Physalacriaceae*	*Armillaria*	+ssRNA and −ssRNA [121,279,280]
			*Flammulina*	dsRNA [281,282]
		*Pleurotaceae*	*Pleurotus*	dsRNA and +ssRNA [270,283,284,285]
		*Pluteaceae*	*Volvariella*	dsRNA [204,286]
		*Porotheleaceae*	*Megacollybia*	dsRNA [204]
		*Psathyrellaceae*	*Coprinopsi*	dsRNA and +ssRNA [204,281]
		*Schizophyllaceae*	*Schizophyllum*	+ssRNA [204]
		*Strophariaceae*	*Agrocybe*	dsRNA [281,287]
		*Squamanitaceae*	*Phaeolepiota*	+ssRNA [204]
	Auriculariales	*Auriculariaceae*	*Auricularia*	dsRNA, +ssRNA and −ssRNA [288,289,290]
	Boletales	*Boletaceae*	*Boletus*	+ssRNA [204]
		*Pisolithaceae*	*Pisolithus*	dsRNA [291]
	Cantharellales	*Cantharellaceae*	*Craterellus*	+ssRNA [204]
		*Ceratobasidiaceae*	*Ceratobasidium*	+ssRNA [292,293]
			*Rhizoctonia*	dsRNA, +ssRNA and −ssRNA [294,295,296,297]
			*Thanatephorus*	+ssRNA [298]
		*Tulasnellaceae*	*Tulasnella*	−ssRNA [218]
	Gloeophyllales	*Gloeophyllaceae*	*Neolentinus*	+ssRNA [204]
	Hymenochaetales	*Hymenochaetaceae*	*Fomitiporia*	+ssRNA [63]
	Polyporales	*Grifolaceae*	*Grifola*	dsRNA [299]
		*Phanerochaetaceae*	*Phlebiopsis*	dsRNA and +ssRNA [300,301]
	Russulales	*Albatrellaceae*	*Albatrellopsis*	+ssRNA [302]
		*Bondarzewiaceae*	*Bondarzewia*	dsRNA and −ssRNA [303]
			*Heterobasidion*	dsRNA and +ssRNA [304,305,306]
		*Russulaceae*	*Lactarius*	dsRNA [121]
	Thelephorales	*Thelephoraceae*	*Thelephora*	dsRNA [204,307]
Exobasidiomycetes	Exobasidiales	*Exobasidiaceae*	*Exobasidium*	dsRNA [304,308,309]
	Tilletiales	*Tilletiaceae*	*Tilletia*	dsRNA [310]
Malasseziomycetes	Malasseziales	*Malasseziaceae*	*Malassezia*	dsRNA [311]
Microbotryomycetes	Sporidiobolales	*Sporidiobolaceae*	*Rhodosporidiobolus*	dsRNA [312]
	Pucciniales	*Coleosporiaceae*	*Cronartium*	dsRNA and +ssRNA [121,313,314]
		*Melampsoraceae*	*Melampsora*	dsRNA [315]
		*Pucciniaceae*	*Puccinia*	dsRNA and +ssRNA [316,317,318,319]
			*Uromyces*	dsRNA [52]
		*Phakopsoraceae*	*Phakopsora*	dsRNA [52]
Tremellomycetes	Cystofilobasidiales	*Mrakiaceae*	*Phaffia*	dsRNA [320,321]
		*Cystofilobasidiaceae*	*Cystofilobasidium*	dsRNA [322]
	*Tremellales*	*Cryptococcaceae*	*Cryptococcus*	dsRNA [323]
	*Trichosporonales*	*Trichosporonaceae*	*Trichosporon*	dsRNA [324]
Wallemiomycetes	Wallemiales	*Wallemiaceae*	*Wallemia*	dsRNA [98]

**Table 3:** Mycoviruses associated with the early diverging lineages of fungi.

Blastocladiomycota				
Class	Order	Family	Genera	Mycoviruses
Blastocladiomycetes	Blastocladiales	*Blastocladiaceae*	*Allomyces*	dsRNA [2,325,326]
**Chytridiomycota**				
Class	Order	Family	Genera	Mycoviruses
Chytridiomycetes	Chytridiales	*Chytridiaceae*	*Zopfochytrium*	dsRNA [2]
	Cladochytriales	*Cladochytriaceae*	*Cladochytrium*	dsRNA [2]
	Rhizophydiales	*Rhizophydiales incertae sedis*	*Operculomyces*	dsRNA and +ssRNA [2]
		*Rhizopodaceae*	*Rhizopus*	dsRNA and +ssRNA [2,327]
**Mucoromycota**				
Class	Order	Family	Genera	Mycoviruses
Glomeromycetes	Archaeosporales	*Geosiphonaceae*	*Geosiphon*	+ssRNA [2]
	Diversisporales	*Gigasporaceae*	*Gigaspora*	dsRNA, +ssRNA and ssDNA [2,52,328]
	Glomerales	*Glomeraceae*	*Glomus*	dsRNA [329]
			*Racocetra*	dsRNA and +ssRNA [52]
			*Rhizophagus*	+ssRNA [2,32,330]
	Paraglomerales	*Paraglomeraceae*	*Paraglomus*	+ ssRNA [52]
Mortierellomycetes	Mortierellales	*Mortierellaceae*	*Dissophora*	dsRNA [2]
			*Lobosporangium*	dsRNA [2]
			*Mortierella*	dsRNA, +ssRNA and −ssRNA [2]
Mucoromycetes	Mucorales	*Choanephoraceae*	*Blakeslea*	dsRNA [2]
			*Choanephora*	dsRNA and +ssRNA [2]
		*Cunninghamellaceae*	*Absidia*	dsRNA [2]
		*Mucoraceae*	*Mucor*	dsRNA, −ssRNA and ssDNA [52,53,331,332]
		*Phycomycetaceae*	*Phycomyces*	[2]
		*Syncephalastraceae*	*Syncephalastrum*	dsRNA [107]
Umbelopsidomycetes	Umbelopsidales	*Umbelopsidaceae*	*Umbelopsis*	dsRNA [2,41,333]
**Neocallimastigomycota**				
Class	Order	Family	Genera	Mycoviruses
Neocallimastigomycetes	Neocallimastigales	*Neocallimastigaceae*	*Anaeromyces*	+ssRNA [2]
			*Neocallimastix*	dsRNA [2]
			*Pecoramyces*	ssDNA [52]
**Zoopagomycota**				
Class	Order	Family	Genera	Mycoviruses
Entomophthoromycetes	Entomophthorales	*Ancylistaceae*	*Conidiobolus*	dsRNA and +ssRNA [2,334,335,336]
		*Entomophthoraceae*	*Entomophaga*	dsRNA [2]
			*Entomophthora*	+ssRNA [2,52]
			*Zoophthora*	dsRNA and +ssRNA [2]
Kickxellomycetes	Kickxellales	*Kickxellaceae*	*Kickxella*	dsRNA and +ssRNA [2]
Zoopagomycetes	Zoopagales	*Piptocephalidaceae*	*Syncephalis*	+ssRNA [2]

**Table 5:** Genomic features and characteristics of + ssRNA mycoviruses.

Families and Genera	Genome Size and Segmentation	Genome Organization	Exemplar Species
Family *Alphaflexiviridae* Mycovirus associated genera *Botrexvirus* *Sclerodarnavirus*	Non-segmented genomes around 5.5 to 9 kb in size.	*Botrexvirus*: Genome consists of up to 5 ORFs. ORF1: Encodes for an RdRp, Hel, and Mtf. ORF3: Encodes for a CP. All remaining ORFs encode for putative proteins with unknown function. *Sclerodarnavirus*: Genome consists of a single ORF encoding for an RdRp, Mtf, and Hel.	*Botrexvirus*: Botrytis virus X *Sclerodarnavirus*: Sclerotinia sclerotiorum debilitation-associated RNA virus
Family *Barnaviridae* Mycovirus associated genera *Barnavirus*	Non-segmented with genomes around 4.0 kb in size.	Genome consists of 4 ORFs. ORF1: Encodes for a hypothetical protein with an unknown function. ORF2: Encodes for a putative serine protease. ORF3: Encodes for an RdRp. ORF4: Encodes for a CP.	Mushroom bacilliform virus
Family *Botourmiaviridae* Mycovirus associated genera *Botoulivirus* *Betabotoulivirus* *Magoulivirus* *Scleroulivirus* *Betascleroulivirus* *Deltascleroulivirus* *Penoulivirus* *Rhizoulivirus* *Betarhizoulivirus*	Non-segmented genomes around 3 to 5.3 kbp in size	Genome consists of a single ORF encoding for an RdRp	*Botoulivirus*: Botrytis cinerea ourmia-like virus 4 *Betabotoulivirus: * Entoleuca ourmia-like virus 1 *Magoulivirus*: Magnaporthe oryzae ourmia-like virus 1 *Scleroulivirus*: Sclerotinia sclerotiorum ourmia-like virus 1 *Betascleroulivirus*: Botrytis cinerea ourmia-like virus 10 *Deltascleroulivirus*: Botrytis cinerea ourmia-like virus 5 *Penoulivirus*: Aspergillus neoniger ourmia-like virus 1 *Rhizoulivirus*: Rhizoctonia solani ourmia-like virus 1Rs *Betarhizoulivirus*: Rhizoctonia solani ourmia-like virus 5
Family *Deltaflexiviridae* Mycovirus associated genera *Deltaflexivirus*	Non-segmented with genomes around 6 to 8 kbp in size	Genome consists of 1–5 ORFs ORF 1: Encodes for polyprotein with Mt, Gtf, Hel and RdRp domains ORF 2–5: Encode for hypothetical proteins with unknown functions	Sclerotinia sclerotiorum deltaflexivirus 1
Family *Endornaviridae* Mycovirus associated genera *Alphaendornavirus* *Betaendornavirus*	Non segmented genomes around 9.5 to 17.6 kb in size.	Genome consists of a single ORF encoding for a large polyprotein. The polyprotein always contains an RdRp domain, but may also include domains for Hel, Mt, Gtf, CPS, and phytoreo S7 domains.	*Alphaendornavirus*: Oryza sativa alphaendornavirus *Betaendornavirus*: Sclerotinia sclerotiorum endornavirus
Family *Gammaflexiviridae * Mycovirus associated genera *Mycoflexivirus*	Non-segmented genomes around 6.8 to 9.2 kb in size.	Consists of 2–3 ORFs ORF 1: Encodes for a replicase (REP) with an upstream Mtf domain, a Hel domain, and a downstream RdRp domain. ORF 2: Encodes for either a CP or proteins which resemble movement proteins. ORF 3: Encodes for either a CP or a hypothetical protein with an unknown function.	Botrytis virus F
Family *Hadakaviridae* Mycovirus associated genera *Hadakavirus*	Multisegmented genomes around 14 to 15 kb in total size.	RNA 1: Encodes for an RdRp. RNA3: Encodes for a Mtf. RNA 2, 4–7, 9 and 10: Encode for hypothetical proteins with an unknown function. RNA8: May encode for a C_2_H_2_-type zinc finger protein	hadaka virus 1
Family *Hypoviridae * Mycovirus associated genera *Alphahypovirus* *Betahypovirus* *Epsilonhypovirus* *Etahypovirus* *Thetahypovirus*	Non-segmented genomes around 9.1 to 12.7 kb in size	Consist of 1–2 ORFs Encodes for a polyprotein containing RdRp and sometimes protease, Hel, and Gtf domains. Some hypoviruses also have short, internally deleted, defective interfering replicative forms of dsRNA molecules, while others have replicative forms of satellite like RNAs	*Alphahypovirus* Cryphonectria hypovirus 2 *Betahypovirus*: Cryphonectria hypovirus 4 *Epsilonhypovirus*: Agaricus bisporus virus 2 *Etahypovirus*: Sclerotium rolfsii hypovirus 8 *Thetahypovirus*: Botrytis cinerea hypovirus 4
Family *Mitoviridae* Mycovirus associated genera *Unuamitovirus* *Duamitovirus* *Triamitovirus* *Kvaramitovirus*	Non-segmented genomes around 2.0 kbp to 4.5 kb in size	Consists of 1 ORF encoding for an RdRp	*Unuamitovirus*: Botrytis cinerea mitovirus 2 *Duamitovirus*: Alternaria alternata mitovirus 1 *Triamitovirus*: Rhizoctonia solani mitovirus 30 *Kvaramitovirus*: Ophiostoma mitovirus 7
Family *Narnaviridae* Mycovirus associated genera *Narnavirus*	Non-segmented genomes around 2.3 to 3.6 kb in size	Consists of 1 ORF encoding for an RdRp	Saccharomyces 20S RNA narnavirus
Family *Yadokariviridae* Mycovirus associated genera *Alphayadokarivirus* *Betayadokarivirus*	Non-segmented genomes around 3.6 to 6.3 kb in size	Genome consists of a 1–2 ORFs encoding for a polyprotein. *Alphayadokarivirus* Consist of 1 ORF which encodes for a polyprotein with an RdRp domain, 2A-like self-cleaving peptide and a hypothetical protein domain with an unknown function. *Betayadokarivirus* Consist of 1 or 2 ORFs encoding for a polyprotein, which may or may not include a 2A-like self-cleaving peptide. The polyprotein contains an RdRp domain (5′ proximal ORF), and a hypothetical protein domain (3′ proximal ORF) with an unknown function.	yado-kari virus 2

Compiled using data from the International Committee on Taxonomy of Viruses (ICTV): https://ictv.global/taxonomy/ (accessed on 27 March 2023). Hamid, et al. [234], Li, Zheng, Cheng, Chen, Fu, Jiang and Xie [235].), Ma, Zhang, Qi, Zhang, Ma, Jiang, Qin and Qi [172], Li, Sun, Yu, Chen, Liu, Yin, Guang, Yang and Mo [295].

**Table 6:** Genomic features and characteristics of—ssRNA mycoviruses.

Families and Genera	Genome Size and Segmentation	Genome Organization	Exemplar Species
Family *Mymonaviridae* Mycovirus associated genera *Auricularimonavirus* *Botrytimonavirus* *Lentimonavirus* *Penicillimonavirus* *Sclerotimonavirus*	Non-segmented genomes around 6 to 10 kb in size	Consists of 1–7 ORFs All mymonaviruses encode for an RdRp near the C-terminus, and some may encode for a NP near the 5′ terminus. Some also encode for hypothetical proteins with unknown functions.	*Auricularimonavirus*: Auricularia heimuer negative-stranded RNA virus 1 *Botrytimonavirus*: Botrytis cinerea negative-stranded RNA virus 5 *Lentimonavirus*: Lentinula edodes negative-strand RNA virus 1 *Penicillimonavirus*: Penicillium adametzioides negative-stranded RNA virus 1 *Sclerotimonavirus*: Botrytis cinerea negative-stranded RNA virus 3

Compiled using data from the International Committee on Taxonomy of Viruses (ICTV): https://ictv.global/taxonomy/ (accessed on 20 March 2023).

**Table 7:** Genomic features and characteristics of RT ssRNA mycoviruses.

Families and Genera	Genome Size and Segmentation	Genome Organization	Exemplar Species
Family *Metaviridae* Mycovirus associated genera *Metavirus*	Non-segmented genomes around 3 to 15 kb in size	Genome consists of an intragenic region which may code for up to 2 genes (gag and pol), which is flanked by long terminal repeat sequences (LTR) The gag gene usually encodes for the CP or NC protein while the pol gene encodes a polyprotein with PR, RT, INT and RH domains	Cladosporium fulvum T-1 virus
Family *Pseudoviridae* Mycovirus associated genera *Hemivirus* *Pseudovirus*	Non-segmented genomes that range in length from 4 to 9 kb in size	Genome consists of an internal region with 1 (gag-pol) or 2 ORFs (gag and pol), which are flanked by long terminal repeat sequences (LTR) The gag protein typically contains the CP and NC domains, while the pol protein contains PR, RT, INT, and RH domains	*Hemivirus*: Candida albicans Tca2 virus *Pseudovirus*: Saccharomyces cerevisiae Ty1 virus

Compiled using data from the International Committee on Taxonomy of Viruses (ICTV): https://ictv.global/taxonomy/ (accessed on 20 March 2023).

**Table 8:** Genomic features and characteristics of ssDNA mycoviruses.

Families and Genera	Genome Size and Segmentation	Genome Organization	Exemplar Species
Family *Genomoviridae* Mycovirus associated genera *Gemycircularvirus* *Gemytripvirus*	*Gemycircularvirus * Monopartite genomes around 1.8 to 2.4 kb in size *Gemytripvirus* Tripartite genome with individual segments around 1.3 kb	*Gemycircularvirus * Genome encodes for a rolling circle REP protein as well as a CP in ambisense orientation *Gemytripvirus* DNA-A: Encodes for the REP protein DNA-B: Encodes for the CP DNA-C: Encodes for p26, a protein with an unknown function	*Gemycircularvirus* Sclerotinia sclerotiorum hypovirulence-associated DNA virus 1 *Gemytripvirus* Fusarium graminearum gemytripvirus 1

Compiled using data from the International Committee on Taxonomy of Viruses (ICTV): https://ictv.global/taxonomy/ (accessed on 20 March 2023), Li, Wang, Zhang, Qiu, Zhou and Guo [175] and Varsani and Krupovic [14].

## Data Availability

No new data was created or analyzed in this study. Data sharing is not applicable to this article.
