# Correction: Hough et al. Fungal Viruses Unveiled: A Comprehensive Review of Mycoviruses. *Viruses* 2023, *15*, 1202

**DOI:** 10.3390/v16040632

**Published:** 2024-04-19

**Authors:** Bianca Hough, Emma Steenkamp, Brenda Wingfield, David Read

**Affiliations:** Forestry & Agricultural Biotechnology Institute (FABI), Department of Biochemistry, Genetics & Microbiology, University of Pretoria, Pretoria 0002, South Africa; bianca.hough@up.ac.za (B.H.); emma.steenkamp@up.ac.za (E.S.); david.read@up.co.za (D.R.)

In the original publication [1], there was a terminology mistake in the sentence “Within this study, the exposure of **plasma** from ALL patients in remission resulted in a significant reappearance of cell surface and genetic markers consistent with this disease [386]” which is in the fourth paragraph of Section 4.2. The corrected sentence should be “Within this study, the exposure of **mononuclear blood cells** from ALL patients in remission **to the supernatant of a mycovirus-containing *Aspergillus flavus*** resulted in a significant reappearance of cell surface and genetic markers consistent with this disease [386]”.

The authors state that while the terminology may be incorrect, the idea behind the paragraph remains the same and the conclusion will thus not be influenced. This correction was approved by the Academic Editor. The original publication has also been updated.
